# Prognostic impact of apoptosis marker Fas (CD95) and its ligand (FasL) on bladder cancer in Egypt: study of the effect of schistosomiasis

**DOI:** 10.3332/ecancer.2012.278

**Published:** 2012-11-06

**Authors:** HM Elmansy, AF Kotb, O Hammam, H AbdelRaouf, HK Salem, M Onsi, T ElLeithy

**Affiliations:** 1 Division of Urology, McGill University Health Centre, Montreal, QC, Canada; 2 Department of Urology, Alexandria University, Alexandria, Egypt; 3 Department of Pathology, Theodor Bilharz Research Institute, Giza, Egypt; 4 Department of Urosurgery, Kasr El-Einy Hospital, Cairo, Egypt; 5 Department of Urology, Theodor Bilharz Research Institute, Giza, Egypt

**Keywords:** apoptosis, Fas, Fas ligand, bladder carcinoma, schistosomiasis

## Abstract

**Objective:**

The Fas–Fas ligand (FasL) system has been recognized as a major pathway for the induction of apoptosis in cells and tissues. Fas-mediated apoptosis is deeply involved in cancer cell death brought about by the immune system. This study was performed to determine the Fas and FasL expression in human bladder cancer and the impact of schistosomiasis infection.

**Material and methods:**

Of the 75 patients, 25 with chronic bilharzial cystitis and 50 with bladder cancer were included in this study. Ten control patients were included in the study, following their consent. Fas and FasL expressions in bladder tissue were determined by indirect immunohistochemistry using avidin-biotin-peroxidase complex (ABC) method.

**Results:**

The association of bilharziasis with malignancy raised the incidence of Fas positive immunoreactivity to 100%. The number of malignant cases positive for Fas decreased with progress of tumour grade and stage. All control cases were negative for FasL expression. The percentage of positive FasL malignant cases increased with increasing tumour grade or stage.

**Conclusion:**

Malignant bladder lesions express high levels of Fas and decreased expression of Fas is associated with disease progression. Urinary bladder carcinoma acquires the functional FasL during tumour progression that may induce apoptosis of anti tumour T lymphocytes. Fas and FasL are recommended to be considered important tumour markers to define aggressive bladder cancer and may be included in defining the surveillance protocol for superficial bladder cancer.

## Introduction

Apoptosis is the genetically mediated mechanism by which individual cells regulate their own deletion [1, 2]. The Fas–Fas ligand (FasL) system has been recognized as a major pathway for the induction of apoptosis in cells and tissues [3]. Fas is present as a cell surface membrane bound receptor and as a soluble protein. Cell-surface receptor (death receptor) is widely expressed in normal tissues (thymus, liver, lung, heart and ovary) and neoplastic tissues; but the expression of this protein does not necessarily predict susceptibility to apoptosis [4].

Fas ligand (FasL or death factor) is expressed mainly in cytotoxic T lymphocytes [5], immune privileged sites [6], and in various tumours [7]. Ligation of Fas to its natural ligand transmits a “death signal” to the target cells, potentially triggering apoptosis [3].

Schistosome-related cancers show different clinical and pathological features compared with non-schistosome-related bladder cancer [8]. Both Bilharzial-related urothelial carcinoma and squamous cell carcinoma of the bladder differ in the expression of apoptosis markers [9]. Cell growth, motility, differentiation and apoptosis are regulated in part by signals received by cell from environment. Cancer cells are characterized by their lack of growth control.

Fas mediated apoptosis is deeply involved in cancer cell death brought about by the immune system. After activation through T cell receptor, cytotoxic T cells express FasL, which binds to Fas on target cells, including cancer cells, to induce apoptosis [10]. FasL is one of the most intensively studied cytokines, a family of proteins that regulate cellular proliferation, differentiation by binding to their specific receptors on target cells [2].

Malignancy may not be associated exclusively with enhanced cell proliferation but may also be linked to decreased cell death [11]. The expression of FasL was originally thought to be restricted to the immune system (activated T-lymphocytes and natural killer cells) where it plays a role in the maintenance of T-cell homeostasis; however, it is now well known that FasL is also expressed by non-lymphoid cells and a variety of non-lymphoid human tumours. This raises the possibility that tumour-expressed FasL induces apoptosis of anti-tumour activated T-lymphocyte representing a novel mode of tumour immune escape [12]. Although predictive factors when considered in combination give some indication of the likely clinical course, there is individual variation in tumour behaviour. Additional prognostic indicators are required to further elucidate which tumours should be treated early in an aggressive fashion and to improve surveillance [9].

The aim of our study was to determine Fas and FasL expression in bladder cancer in Egypt and the role of associated Schistosomiasis infestations.

## Materials and methods

We conducted a prospective study of 75 consecutive individuals who presented to the Theodor Bilharz Research Institute (TBRI), Cairo, Egypt. Ten healthy adults with normal healthy urothelium were added as a control group. The control group was taken consecutively, from the patients who underwent cystoscopic procedures for different causes such as ureteric stenting, and tested negative for Schistosoma antibodies in their serum and also tested negative for both Schistosoma eggs and malignancy after cystoscopy and biopsies (after written consent) were performed. Detailed history, clinical examination, routine laboratory investigations, diagnosis of schistosomal infestation by detection of Schistosoma eggs in tissues and/or detection of Schistosoma antibodies in sera of patients by enzyme immunoassay, urine cytology, ultrasonography (abdomen and pelvis), intravenous urography, cystoscopy with transurethral resection of any visible mass or biopsy from any suspicious area, plus random biopsies from all bladder walls, and histopathological assessment were done. A histopathological diagnosis was carried out using the criteria and classification of urothelial tumours as outlined by Eble *et al* [13]. According to the above-mentioned investigations, the 75 patients were categorized into the following groups: Chronic Bilharzial Cystitis (25 cases), urothelial carcinoma (UC) group (31 cases) including 6 cases UC with bilharziasis and 25 cases non-bilharzial UC and Squamous cell carcinoma (Sq CC) group (19 cases). All of the 19 cases were bilharzial associated.

The TUR biopsies were fixed immediately after removal in 10% formalin buffered with 0.1 M phosphate buffer. Tissue section slides were stained by a haematoxylin and eosin (H&E) stain and examined by light microscopy for histopathologic examination and morphologic evaluation of the disease. Assessment of Fas and FasL in tissue was done by immunohistochemistry [14] using rabbit polyclonal anti human Fas & FasL antibody and detection kit DAKO SAB2 System, HRP (DAB) (Code no. C-20) (Santa Cruz Biotechnology, Inc., USA). After a quick rinse in phosphate-buffered saline (PBS), two sections were covered with the primary antibody (Rabbit anti-Fas & FasL polyclonal IgG) and were applied in a humid room temperature overnight in a dilution of 1:40. The sections were then incubated for 30 min with the secondary biotinylated antibody followed by avidin peroxidase complex for another 30 min according to the manufacturer’s instructions (Universal Detection Kit, Dako, Denmark). The brown colour was developed using diaminobenzidine (DAB) for 2–4 min, washed in distilled water and counterstained with Mayer’s haematoxylin for 1 min. The entire procedure was performed at room temperature. In addition, negative controls in which the primary antibody was omitted and replaced by PBS were also used. The expression of Fas and FasL were measured in 10 successive high power fields (×400). Fas and FasL showed mostly cytoplasmic expression with condensation on the nuclear membrane. The percentage of positively stained cells was determined semi-quantitatively by assessing the whole tumour section, and each sample was assigned to one of the following categories: Intensity of staining was graded on a scale of 0, +1, +2, and +3 with 0 represented no detectable stain and +3 represented the strongest stain [15]. The mean number of cells positive for Fas & FasL staining was calculated.

Statistical analysis was performed using SPSS 9 software program. Statistical presentation and analysis of the present study were conducted using the mean (*x*), standard deviation (SD) between two variants applying the Student *t* test, and for more than two variants we use the ANOVA test. The correlation between Fas & FasL tissue expression, histopathological stage grade, and clinical data were done using the χ2 test.

## Results

The highest frequency in cystitis and malignant cases was in the seventh decade of age. The mean age was 57 ± 12.35 and 49 ± 10.02 years for malignant and cystitis cases, respectively. Table 1 shows the age and sex distribution of the studied groups. Fas positivity was seen in 60% of control cases, all were of mild intensity. All control cases were negative for FasL immunoreactivity. In the study groups, 64% out of chronic schistosomal cystitis showed positive Fas immunoreactivity and 44% showed positive FasL immunoreactivity. The development of malignancy raised the incidence of positive cells to 92 and 82% for Fas and FasL, respectively.

When malignant cases were classified according to their schistosomal infestation, we found that bilharzial infestation is associated with invasiveness of the bladder tumour, whereas in the bilharzial malignant cases, 72% had invasive tumours (stages T2 and T3) and 64% were of high grade (grades II and III), while in the non-bilharzial cases, invasive tumours and high-grade tumours represented 48 and 60%, respectively. Considering Fas and FasL immunoreactivity, bilharzial-associated lesions had 100 and 92% Fas and FasL positivity, respectively, while non-bilharzial lesions had 84 and 72% positivity, respectively.

If we reclassify the malignant lesions according to the pathological type, all SqCC cases showed Fas and FasL positive immunoreactivity, while only 87 and 71% of UC showed Fas and FasL positivity, respectively. Table 2 illustrates the extent of Fas and FasL immunoreactivity in the studied groups. Table 3 shows the extent of Fas and FasL immunoreactivity within the malignant cases.

Correlating Fas and FasL immunoreactivity with the tumour grade and stage, we found that increasing tumour grade and stage is associated with a decrease in Fas and increase in FasL immunoreactivity. Table 4 shows the analysis based on histological grade and stage.

**Figure 1:**
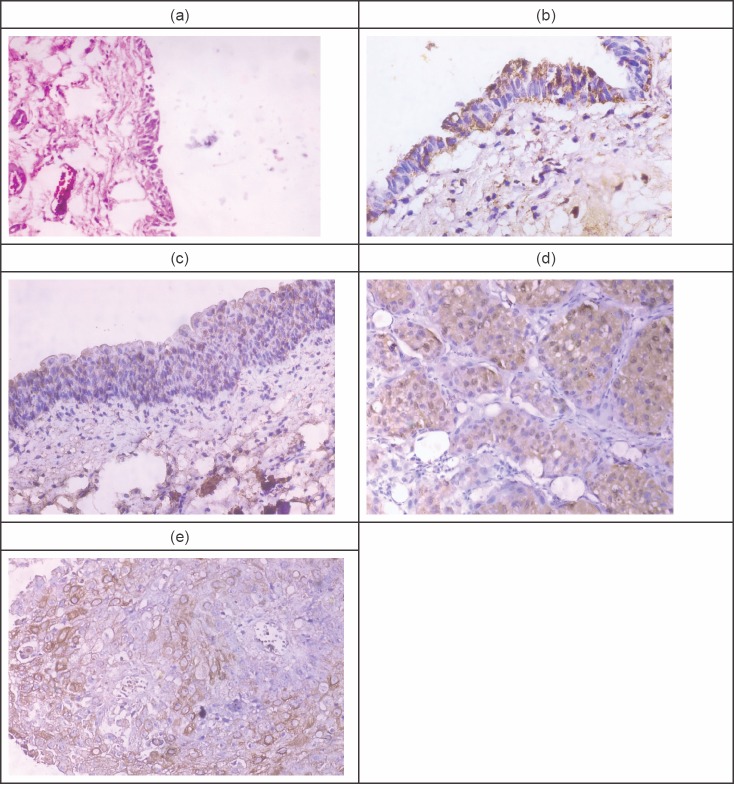
Fas and FasL immunoreactivity in normal, chronic cystitis, and malignant cases. (a): Normal urothelium (H & E. X100). (b): Normal urothelium, showing mild Fas immunoreactivity (X400). (c): A case of Sch. cystitis, showing moderate Fas immunoreactivity (X200). (d): A case of SqCC showing moderate FasL immunoreactivity (X400). (e): A case of UC showing moderate FasL immunoreactivity (X400).

Figure 1 illustrates Fas and FasL expression in normal urothelium, chronic cystitis, and malignant cases. It shows the normal urothelium with preserved umbrella cells, then mild Fas immunoreactivity that appears as a brownish intracytoplasmic staining. Panel c represents moderate Fas immunoreactivity in 50% of urothelial cells in a chronic cystitis case. Panel d represents an example of moderate FasL immunoreactivity in a case with SqCC, while panel e represents moderate FasL immunoreactivity in a case with urothelial carcinoma.

## Discussion

The death receptor Fas (Apo1/CD95) and FasL system is recognized as a major pathway for the induction of apoptosis in vivo and anti-apoptosis via its blockade that plays a critical role in carcinogenesis and progression in several malignancies. However, the function of Fas–FasL system in urothelial cancer has not been elucidated [4].

Fas positivity was seen in 60% of control cases, all of mild intensity, and stained the cell membrane and the cytoplasm; this is in agreement with Yamana *et al* [4] who found Fas widely expressed in healthy adults, in about 92% of cases.

When patients were stratified according to their schistosomal infestation, we found 64% of schistosomal chronic cystitis cases showed positive Fas immunoreactivity. This increase may be attributed to the pathological changes induced by schistosomal infestation as metaplasia and dysplasia.

In our work, development of malignancy raised the incidence of the Fas positive cells to 92%. Fas positivity in non-bilharzial malignant lesions was 84%, whereas Fas positivity in bilharzial-associated malignant lesions was 100%; so the association of bilharziasis with malignancy raises the incidence to be 100%. All SqCC (100%) showed Fas positive immunoreactivity, whereas 87% of UC showed Fas positivity.

Analysis of the histological stage and grade of bladder cancer revealed that the number of malignant cases positive for Fas decreased from 94.7 to 91.7% and 85.7% with progress of malignancy grade from grade I to grades II and III, respectively. The number of malignant cases positive for Fas decreased from 95 to 91.6% and 83.3% with progress of malignancy stage from stage I to stages II and III, respectively. This finding is in accordance with Lee *et al* [7] who demonstrated Fas and FasL expressions in the UC by immunohistochemistry. The UC showed immunoreactivity for Fas in 41 of 43 cases (95%). The two Fas-negative TCCs were a grade II/stage T2 tumour and a grade III/ stage T3 tumour. FasL was expressed in all TCCs analyzed (100%). Fas and FasL immunostainings, when present, were cytoplasmic and along the cell membranes; nuclei were clearly negative. All TCCs with the Fas gene mutations co-expressed Fas and FasL.

In a study done by El Baz *et al* [16], the malignant cases showed a highly significant increase in the mean percentage of expression of Fas in tissue compared to the chronic cystitis (*P* < 0.001) group. There was an increase in the percentage of expression of Fas in the tissue of the invasive group compared to the non-invasive group, with no significant difference between both groups. However, Fas expression was significantly higher in grade II than in grade III.

Mass *et al* [17] reported that Fas down-regulation was related to higher tumour stage, which is the same as our results. Among Fas-related molecules, Fas and its absence may have the greatest impact on tumour progression through evading apoptosis leading to a poorer progression. Thus, the loss of Fas was suggested to be a crucial factor for selecting patients requiring more aggressive treatment. Recent studies showed that decreased expression of Fas is associated with disease progression.

In our study, all control cases were negative for FasL immunoreactivity. 44% out of chronic schistosomal cystitis showed positive FasL immunoreactivity. 82% carcinoma cases showed positive FasL immunoreactivity.

Non-bilharzial malignant lesions showed FasL positivity of 72%. The presence of bilharziasis raises the incidence to be 92%. 71% of TCC cases showed FasL positive immunoreactivity. All SqCC cases showed FasL positive immunoreactivity.

All grade III carcinoma, 83.3% of grade II tumours, and 73.7% of grade I tumours showed FasL positive immunoreactivity. Therefore, the presence of positive FasL immunoreactivity increases with the upgrading of tumours. All T3 carcinoma showed FasL positive immunoreactivity. The percent of positive cases decreased with T2 and T1 to be 83.3 and 75%, respectively. Therefore, the percentage of positively stained malignant cases increased with the increasing of the stage. In TCC, less frequent Fas expression was significantly associated with a higher pathological grade (*P* < 0.0001), a more advanced stage (*P* < 0.023), and poorer prognosis (*P* < 0.01). Fas and its absence therefore were suggested to be crucial factors by which we can select patients requiring more aggressive treatment.

Chopin *et al* [12] showed that human urinary bladder TCC acquires the functional FasL during tumour progression. FasL expression was observed in situ in 45% of TCC and was absent in normal urothelium. A correlation existed between FasL expression and high-tumour grade (0% in G1, 14% in G2, 75% in G3, *P* < 0.001) and stage (13% in superficial Ta-T1 tumours versus 81% in invasive T2-T4 tumours, *P* < 0.001). These results suggest that TCC expressed FasL may induce apoptosis of anti tumour T lymphocyte in vivo, providing new insights on the mechanism involved in bladder TCC Progression. All the invasive TCCs associated with a metastatic disease were positive for FasL immunostaining. So, there is a strong correlation between FasL expression by bladder TCCs and the pathological grade and stage of the tumour.

Lee *et al* [7] showed that TCC expresses high levels of FasL in vivo. The expression of FasL in TCC suggests that FasL may contribute to the immune escape through killing Fas bearing lymphocyte.

The expression of Fas with FasL also suggests that TCC may have pathways resistant to Fas-mediated autocrine cell suicide.

T cells immediately adjacent to FasL expressing tumour cells were undergoing apoptosis, where T cells not close to tumour cells were viable, indicating that tumour cells could be responsible for the induction of programmed cell death in the peritumoral T-cells.

In this study, most of the TCC samples (92%) showed Fas immunoreactivity in tumour cells and most (92%) co-expressed Fas and FasL. These results indicate that although TCC expresses Fas on their surface, the TCC may have protective mechanisms against attack from FasL bearing lymphocytes and tumour cells.

Because Fas is widely expressed in normal tissues, FasL expression in TCC might facilitate tumour invasion by triggering apoptosis in the surrounding Fas positive tissues, allowing the tumour to spread locally.

Mehmut *et al* [18] showed that there is a significant increase in the infiltration of FasL after BCG instillation in superficial bladder carcinoma. Moreover, Fas expression was up regulated on tumour cells after BCG instillation, which may play an important role in the therapeutic effect of BCG.

All these data are in agreement with our results that strongly implicate Fas and FasL in the pathogenesis of bladder cancer progression.

Also, 11 out 25 chronic bilharzial cystitis cases (44%) showed the FasL immunoreactivity, almost equally of mild and moderate intensity.

This is important in comparison to control groups which were all negative for FasL immunoreactivity showing the importance of this ligand in the pathogenesis and progression of this disease and in the progression of associated neoplasia, as the presence of Bilhariziasis was associated with increased expression of both Fas and FasL.

## Conclusion

Schistosomiasis has a positive effect on Fas & FasL. Malignant bladder lesions express high levels of Fas and decreased expression of Fas is associated with disease progression. Bilharzial-associated cancer (SqCC or UC) shows higher co-expression of Fas and FasL. Urinary bladder carcinoma acquires the functional FasL during tumour progression that may induce apoptosis of anti-tumour T lymphocytes and is suggested to be a crucial factor for selecting patients requiring more aggressive treatment and may be a crucial test to integrate into the surveillance protocol for patients with superficial bladder cancer, especially if refractory to BCG, augmenting the decision to early aggressive treatment. “augmenting the decision to” should be “augmenting the decision for”.

## Figures and Tables

**Table 1.** Age and sex distribution of the studied group.

	Chronic cystitis	Malignancy
Sex (no.)		
Male	24 (96%)	39 (78%)
Female	1 (4%)	11 (22%)
Age		
Mean	49 ± 10.02	57 ± 12.35
10 – 20	0	2
21 – 30	4	0
31 – 40	5	1
41 – 50	0	9
51 – 60	5	11
61 – 70	11	17
71 – 80	0	10

**Table 2.** Extent of Fas and FasL expression in studied groups.

	Control	Chronic Bil. cystitis	Malignancy	*P* value
Fas (positive cases)	60%	64%	92%	0.001
Fas (mean positive cells)	5 ± 0.75	20.0 ± 13.2	52.2 ± 23.15	0.001
FasL (positive cases)	0%	44%	82%	0.001
FasL (mean positive cells)	0.0 ± 0.0	14.4 ± 9.15	44.2 ± 24.66	0.01

**Table 3.** Extent of Fas and FasL expression in malignant cases.

		Fas	*P* value	FasL	*P* value
Positive cases	Non Bil.(25)	84%	0.01	72%	0.001
	Bil. (25)	100%		92%	
	UC (31)	87%	0.01	71%	0.01
	SqCC (19)	100%		100%	
Mean positive cells	Non Bil. (25)	49.6 ± 19.86	0.05	38.8 ± 28.18	0.01
	Bil. (25)	54.8 6 ± 23.29		49.6 ± 19.68	
	UC (31)	45.48 ± 26.68	0.01	37.48 ± 27.56	0.01
	SqCC (19)	63.15 ± 12.04		55.26 ± 13.48	

**Table 4.** Fas and FasL expression of different studied malignant grades and stages.

	Fas		FasL	
	Positive cases (%)	Mean positive cells	Positive cases (%)	Mean positive cells
Grade 1	94.7	55.78 ± 20.36	73.7	50.52 ± 19.28
Grade 2	91.7	50.41 ± 26.78	83.3	42.08 ± 26.53
Grade 3	85.7	48.57 ± 23.4	100	34.28 ± 29.92
T1	95	55 ± 26.6	75	50.01 ± 18.2
T2	91.6	52.3 ± 22.6	83.3	43.75 ± 29.1
T3	83.3	34.33 ± 23.3	100	26.66 ± 15.05
